# Diffusion model-based understanding of subliminal affective priming in continuous flash suppression

**DOI:** 10.1038/s41598-021-90917-w

**Published:** 2021-06-01

**Authors:** Minchul Kim, Jeeyeon Kim, Jaejoong Kim, Bumseok Jeong

**Affiliations:** 1 Graduate School of Medical Science and Engineering (GSMSE), Korea Advanced Institute of Science and Technology (KAIST), 291 Daehak-ro, Yuseong-gu, Daejeon, 34141 Republic of Korea; 2 Department of Radiology, Kangbuk Samsung Hospital, Sungkyunkwan University School of Medicine, Seoul, Republic of Korea

**Keywords:** Neuroscience, Psychology

## Abstract

Affective states influence our decisions even when processed unconsciously. Continuous flash suppression (CFS) is a new variant of binocular rivalry that can be used to render the prime subliminal. Nonetheless, how prior information from emotional faces suppressed by CFS influences subsequent decision-making remains unclear. Here, we employed a CFS priming task to examine the effect of the two main types of information conveyed by faces, i.e., facial identity and emotion, on the evaluation of target words as positive or negative. The hierarchical diffusion model was used to investigate the underlying mechanisms. A significant interaction effect on response time was observed following the angry face prime but not the happy or neutral face primes. The results of the diffusion model analyses revealed that the priming effects of facial identity were mapped onto the drift rate and erased the ‘positive bias’ (the processing advantage of positive over negative stimuli). Meanwhile, the positive emotional faces increased the nondecision time in response to negative target words. The model-based analysis implies that both facial identity and emotion are processed under CFS.

## Introduction

Emotional and affective processing imposes itself over cognitive processes and modulates our decision-making^1,2^. This emotional influence on human decision-making has been revealed by the affective priming task^3^, which examines the implicit affective association between an emotion prime and target words. In a typical affective priming task, the prime and the target are presented sequentially, and participants are instructed to indicate the valence of the target as quickly as possible. If participants categorize the target faster when it is valence-congruent with the prime (e.g., the prime and the target are both positive) than in the valence-incongruent case (e.g., the prime is positive while the target is negative), the reaction time difference between the congruent and incongruent conditions is called the ‘affective priming effect’.

Previous studies have shown that people evaluate targets based on primed affective information, and this emotion-induced bias was shown to be particularly prominent when the prime was rendered invisible because it escapes regulation by conscious awareness^4–6^. Traditionally, briefly presented primes with forward/backward noise patterns are used to mask the prime from conscious awareness^7^. Continuous flash suppression (CFS), a variant of binocular rivalry and flash suppression, is a relatively new method that has several advantages in making a prime subliminal. The CFS technique presents the stimulus that is to be tested to one eye, but the test stimulus is strongly suppressed by a train of dynamically high-contrast masks that are presented to the other eye^8^. One notable difference between traditional masking and CFS is the duration of subliminal presentation that can be sustained: masking can render primes invisible for tens of milliseconds, whereas CFS can suppress primes from being perceived for seconds^8,9^. This longer suppression time provides an opportunity to test whether invisible stimuli can be integrated into higher-level information^6,10^. Recent studies suggest that not only low-level (i.e., contrast, spatial frequency, and orientation) but also high-level information (i.e., facial information such as identity and emotion) can be processed under CFS^2,6^. For example, Yang et al*.* used an emotional face prime rendered invisible under CFS and a visible emotional target word to test whether high-level information rather than low-level properties can be extracted under CFS and found that a congruent meaning of facial expressions facilitated emotional judgments of subsequent words. They argued that since faces and words have minimal overlapping features, the possibility of perceptual facilitation by low-level features could be ruled out^6^. However, how subliminal facial information perceived under CFS is processed and whether it biases people’s judgment remains unclear.

**Figure 1:**
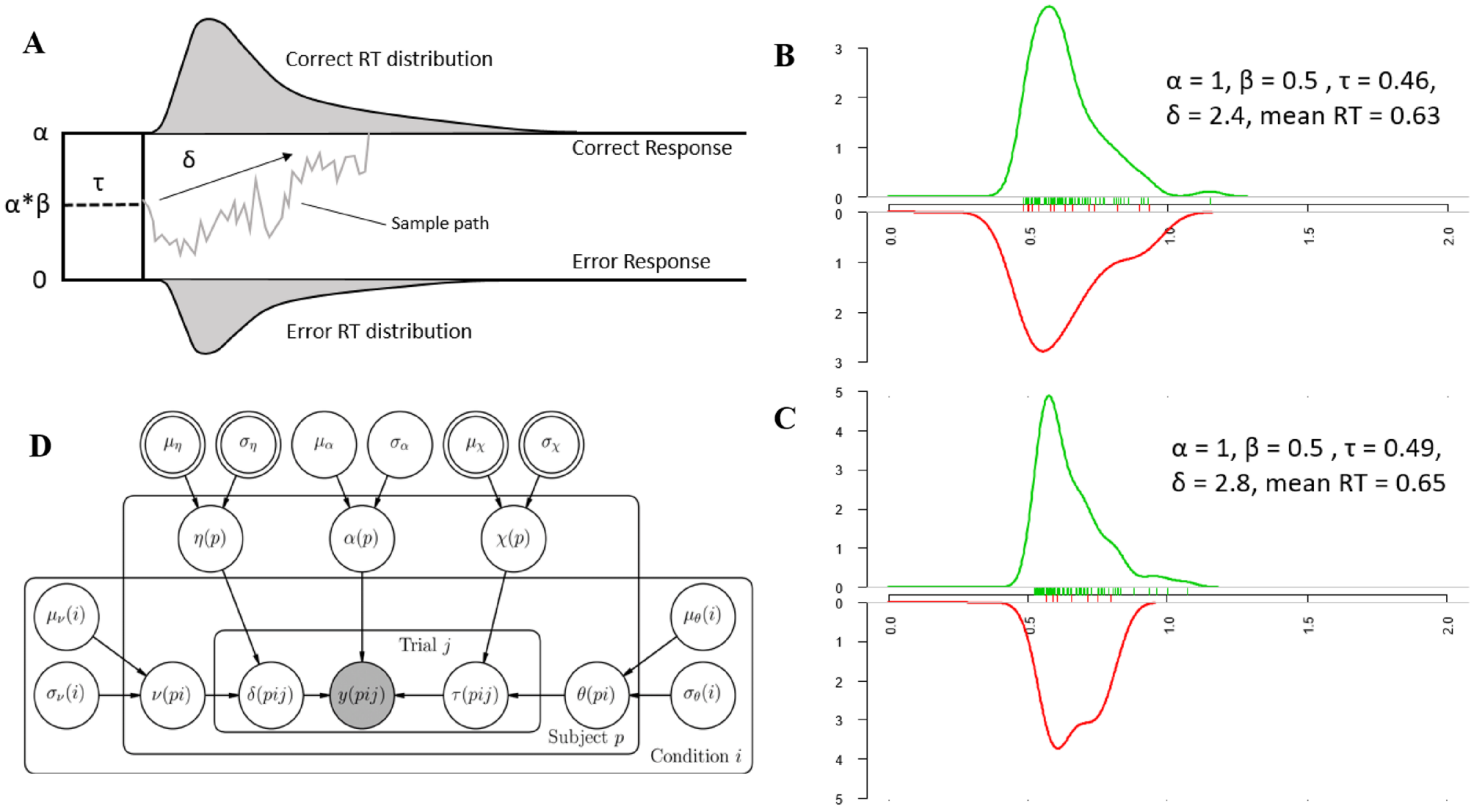
Graphical illustration representing the Wiener diffusion model and the hierarchical diffusion model (HDM). (**A**) A graphical illustration of the Wiener diffusion model. α = boundary separation indicating the evidence required to make a response; β = initial bias indicating the a priori status of the evidence counter as a proportion of α; δ = average rate of information uptake; τ = time used for everything except making a decision. The picture is drawn based on Vandekerckhove et al*.*^15^. (**B**, **C**) Plots showing generated reaction times (RT) via the ‘RWiener’ package^16^ using the parameters shown in each figure. Although different parameters were used to generate the data, the mean reaction times are similar (two-sample t-test; *t* = 1.02, *p* = 0.31). (**D**) Graphical notation of the model used in parameter estimation. We followed the notation method suggested in the book ‘Bayesian Cognitive Modeling’^17^. The node distinctions between observed versus unobserved variables use shaded and unshaded nodes, and those between stochastic versus deterministic variables use single- and double-bordered nodes. Subscripts and plates indicate repetitions of the parameter across participants p, conditions i, and trials j.

We used the diffusion model to investigate the underlying mechanisms of how facial information from a prime influences people’s decisions. The diffusion model, initially proposed by Roger Ratcliff^11^, is a well-developed cognitive model that accounts for the time course of human decision-making in two-choice tasks^11^. The diffusion model conceptualizes a decision between two choices based on the accumulation of evidence favoring one of the decision alternatives^12^. When a participant is asked to categorize the target, the evidence from the target is accumulated over time until it hits an upper or lower boundary. This drift process is characterized by four parameters (Fig. 1A): the initial bias toward one of the alternatives (β), the total time devoted to general, nondecision-related processes (τ, which includes perceptual encoding and motor preparation), the drift rate (ν), and the distance between decision boundaries (α)^13^. If a participant’s reaction time (RT) is high in an experimental condition, we can determine why this is so based on these parameters. Aggregate measures, such as the mean RT, are not sensitive to this complexity, and analyzing only the RT may obscure any relationship with crucial processes that are actually at play during the tasks^14^. Additionally, even when the RTs are similar, a diffusion model analysis can reveal the mechanisms that contribute to the results (Fig. 1B,C).

Using the diffusion model, we focus on testing our hypothesis about the effect of facial information on subsequent decisions. Faces that express emotion (emotional faces) convey the following two main types of information: facial identity and expression^18^. These two types of information have been suggested to have separate posited functional and anatomical routes when a face is perceived^19–21^. Here, we use the term ‘facial identity’ to refer to invariant facial properties, such as an upright intact facial structure or configuration facilitating the recognition of a face, in contrast to changeable facial expressions, such as lip movements that express emotions and facilitate social communication^22^. We aimed to explore whether the two types of information present in subliminal emotional faces are mapped to different parameters. To dissociate the effects of facial identity and facial emotion, we used the following four types of facial primes: scrambled faces that do not have facial identity or emotion as reference stimuli and three types of faces showing positive, negative and neutral emotions. We presumed that each of these three emotional faces has a facial identity and positive, neutral and negative facial expressions. We selected personality adjectives as target words to facilitate a semantic relation to the perceived faces. Based on previous studies, we hypothesized that two candidate parameters would be found to be responsible for the subliminal facial information suppressed under CFS. The first is the drift rate parameter. Voss et al*.* conducted a series of experiments and used the diffusion model to show that the semantically associative (i.e., prime: king, target: crown) priming effects mapped onto the drift rate parameter, indicating that semantic associations facilitate information uptake^23^. The associative network model suggests that the activation of the semantic representation of the prime increases the activation level of the semantic representation of the target^3,24^. In a study investigating the associative priming effect of related word–face pairs, a study found that drift rate accounted for both identity and the associative priming effect^25^. Based on these reports, we hypothesize that drift rate accounts for the association effect between facial identity and personality adjectives, in contrast to the lack of association observed for scrambled face primes. Another possible candidate parameter is the nondecision time. One study found that affective priming with words moderated nondecision time^23^. Additionally, Yap et al*.* reported that when stimuli were degraded, the semantic priming effect mapped onto both the drift rate and the nondecision time^26^. Thus, based on the previous literature, we hypothesized that the priming effect of facial emotional information would mainly map onto the nondecision time.

## Materials and methods

### Participants

To determine the sample size, we consulted several relevant studies that tested congruent priming effects under CFS^6,27^. Almeida et al. reported average standardized effect sizes of d_z_ = 0.58 and 0.51^14^; these values can be transformed to Cohen’s *f* = 0.29 and 0.25. Using G*Power 3.1^28^, we determined that for the given effect sizes and a type I error probability of *α* = 0.05, a sample size of 18–25 is required to achieve a power of 0.80 using the default parameters (repeated-measures analysis of variance (ANOVA) within factors).

A total of 31 individuals (21.5 ± 2.5 years old; 15 males) were recruited via a web-based message board. All participants had normal or corrected-to-normal vision and reported having no ophthalmic disease or convulsions, which could impair the preciseness of the results. All participants provided written consent and received monetary compensation for their participation. The participants were instructed not to consume caffeine-containing drinks or cigarettes from one hour before the experiment. The study was approved by the Korean Advanced Institute of Science and Technology Institutional Review Boards in accordance with the Declaration of Helsinki.

### Stimuli and design

The instructions and stimuli were displayed on a Qnix 24-in. LCD monitor (75 Hz). The participants viewed the stimuli through a Geoscope mirror stereoscope at a distance of approximately 60 cm with their head position fixed. The participants performed the tasks in a dimly lit, soundproof chamber. The affective stimuli consisted of seventy face images (half male) with happy, angry or neutral facial expressions from the Karolinska Directed Emotional Faces dataset^29^. Happy and angry emotions were chosen from among the available other facial expressions because these two emotions were considered adequate to examine the pure effect of valence and because angry faces were found to modulate decisions under CFS in a previous study^2^. All images were transformed into ellipse-cut grayscale images that included only internal features with low contrast and luminance by MATLAB, using the ‘imadjust’ function with the parameters [0 1] and [0.2 0.5]. This procedure maps intensity values in an image to new values such that values between 0 and 1 map to values between 0.2 and 0.5, resulting in a mean brightness of 77 and a mean Michelson contrast of 0.41. The contrast of the faces remained the same over time. The scrambled face images, which were used as the reference stimuli, were created from the processed grayscale images using the ‘randblock’ function in MATLAB (we divided the image into nonoverlapping blocks 5 × 5 pixels in size and shuffled these blocks; the code used to create these images is available at https://osf.io/zqnbt/). The CFS stimuli were Mondrian pattern masks refreshed at a rate of 10 Hz generated using the PsychoPy toolbox, and the size of the Mondrian components on the screen was 1° (https://perso.univ-lyon2.fr/~brogniar/notes/psychopy-continuous-flash/#head.flash_colors.exp). All stimuli were presented surrounded by a black frame to facilitate binocular fusion, and two stimuli subtended approximately 9.5° of visual angle (viewing distance = 60 cm; stimuli frame size = 10 cm, separated by 10 cm; mask and target size (in visual angle) 9.5° × 9.5° and 4.8° × 4.8°, respectively).

In the valence categorization task, 80 personality adjectives (half positive, half negative) were used as target words. Since faces and words have minimal overlapping features, the possibility of perceptual facilitation (i.e., low-level properties) could be ruled out^6^. The target words were three- or four-syllable Korean words selected from a pool of ninety words based on a survey conducted in a separate group (N = 30). The level of valence and the number of syllables were matched between positive and negative words (*t* = 0.30, *p* = 0.76, mean valence rate of positive and negative words = 1.78 and 1.76, respectively, chi-square test for number of syllables: χ^2^ = 0.818, *p* = 0.366).

### Procedure

The eye dominance of each participant was measured using the hole-in-the-card method. The participants were instructed to fix the eye gaze at the center of the stimuli. A central cross was presented to each eye except when the face stimuli and target words were displayed.

**Figure 2:**
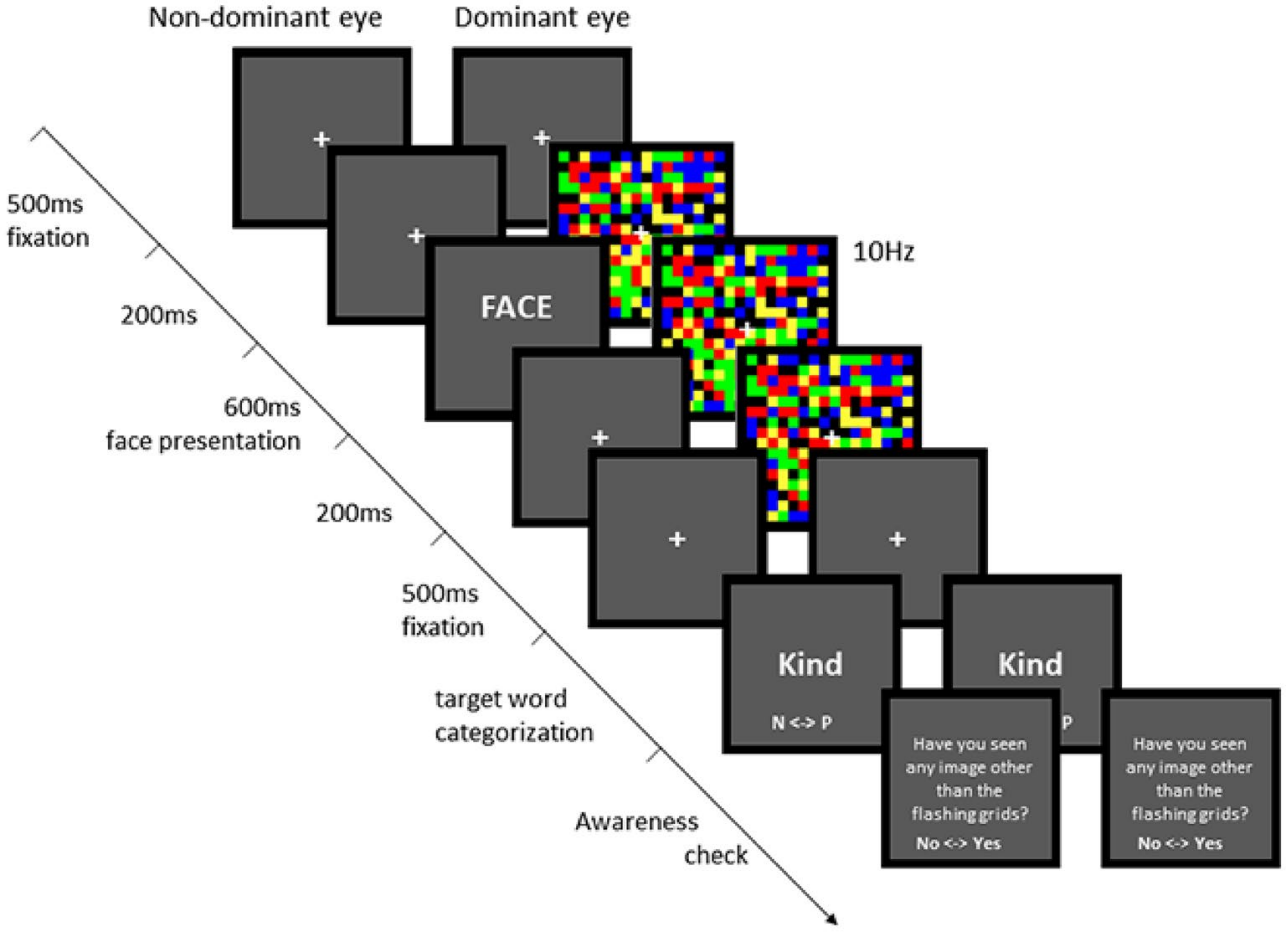
Schematic representation of the experimental trial structure. Following a fixation cross to both eyes, colorful flash grids were presented concurrently with face stimuli, inducing continuous flash suppression (CFS). Word categorization and awareness checks were then performed in sequence.

Figure 2 shows the experimental design of the nonconscious affective priming task (NAPT). In a given trial, the participants were presented with a 500-ms fixation cross surrounded by black square frames to both eyes. Following fixation, high-contrast colorful grids refreshed at a rate of 10 Hz were presented to the participant’s dominant eye for 1000 ms to keep the face prime invisible. Concurrently, a low-luminance, low-contrast grayscale face prime was presented to the participant’s nondominant eye for 600 ms. To enhance suppression by CFS, the face prime was presented 200 ms after the onset of colorful grids and was removed 200 ms before the offset of the colorful grids. Following the priming step, a fixation point was presented to both eyes for 500 ms. The target word was then presented at the center of each visual field to both eyes. The participants were instructed to categorize the word as either positive or negative as quickly and accurately as possible. Speed and accuracy were equally emphasized. It was also emphasized to not ponder the meaning of the word. The participants responded by pressing one of two buttons using their dominant hand. The two button keys were the left and right direction keys on the keyboard, in which the left and right direction keys indicated ‘negative’ and ‘positive’, respectively.

To check whether participants perceived the suppressed faces, they were asked to indicate whether they saw any perceptible image other than flashing colorful grids. As soon as the word categorization response was registered, the text “Have you seen any image other than the flashing grids?” was presented with no/yes option below. The participants responded by pressing either the left or right direction button keys indicating ‘no’ or ‘yes’, respectively.

Three hundred and twenty trials, divided into four blocks, were presented to each participant, yielding 40 trials per condition (4-by-2 within-subject design). Each block contained twenty happy, angry, neutral, and scrambled face stimuli and forty positive and negative words to yield eighty trials. One block took 4–5 min depending on the participant. To ensure that the results were not affected by familiarity with the task, each participant completed 20 practice trials before starting the main task. Between the blocks, the participants were asked to rest for at least two minutes to reduce the possibility of the CFS breaking^30^. The participants were provided with disposable artificial tears to prevent eye drying. The total experiment time for the NAPT, including the break time, was within ~ 30 min.

To check the participants’ knowledge of the target words, the participants completed an evaluation survey with the target words based on two criteria. First, they indicated how much they understood the meaning of the word on a 4-point scale from ‘very well’ (4) to ‘don’t know at all’ (1). Second, they categorized the word into one of the three categories: positive, negative and cannot judge.

### Data analysis

#### Pre-analysis data exclusion

Trials in which participants responded to perceived images (aware trials) were excluded from the analysis^4,6^. Next, we excluded participants who did not complete a sufficient number of within-subject trials (at least 30 trials per condition). To identify outliers, trials with log-transformed RTs that were more than two standard deviations from the mean based on individual RT-distributions were excluded for both unaware and aware trial analyses, as were trials with RTs faster than 200 ms and slower than 5000 ms^12^.

Trials that contained words that the participants did not know the meanings of (where the participants checked ‘2’ or ‘1’ on a 4-point scale) were excluded from the analysis because including such trials would have interfered with automatic word categorization. For trials in which the participants reported knowing the meaning of the particular target word but the meaning was opposite to what they thought, the conditions of the analysis were modified to reflect the participant’s original implicit association for those words.

#### Analysis of reaction times

Since the raw RTs obtained in simple decision tasks are skewed, we used an inverse Gaussian generalized linear mixed-effects model with the identity link function^31^. A sequence of nested models was built in which the random-effects structure showed increasing complexity, with main effects and interactions for face (i.e., scrambled, neutral, happy, and angry) and valence (positive and negative target words) as fixed factors. We attempted to run a model with random intercepts for the combinations of face, valence and subjects. The random slopes for the two main effects were not included because under those conditions the model did not converge. Likelihood ratio tests using the ‘anova’ function showed that the model with the highest goodness of fit is random intercepts for subjects. We used the ‘lme4’ package in the R program for statistical computing^32,33^. Fixed effects were tested for significance using Type III Wald chi-square tests^34^. The R code is expressed as:

$$\begin{aligned} {\text{'glmer}} & \left( {{\text{Reaction}}\;{\text{time}}\sim {\text{face}} + {\text{valence}} + {\text{face:valence}} + \left( {{\text{1|person}}} \right),{\text{data}} = {\text{df }},{\text{family}}} \right. \\ & \quad = {\text{inverse}}.{\text{gaussian}}({\text{link}} = {\text{''identity'')'}} \\ \end{aligned}$$

#### Hierarchical diffusion modeling

The RT and accuracy data were fitted using a hierarchical diffusion model (HDM)^15^ that incorporated fixed effects for faces-by-valence conditions (eight conditions) for estimation of the mean of the drift rate (ν) and the mean of the nondecision time (θ). Hierarchical models are ideally suited to handle data sets with few trials per participant^15^. We allowed the nondecision time τ and the drift rate δ to differ between persons p, conditions i, and trials j. In other words, the drift rate parameter δ (pij) was cast as a random variable with a condition-by-person specific mean ν, and the nondecision time τ (pij) followed the condition-by-person-specific mean θ. These two parameters were chosen for inclusion in our hypothesis based on the previous literature presented in the introduction. In addition to fixed effects, the hierarchical model allows participant-level random effects for boundary separation (α), and we set the bias parameter (β, the relative starting point of the diffusion process between the two boundaries) to 0.5 assuming an unbiased diffusion process following Vandekerckhove et al.^13,15^. We fitted the diffusion model with within-trial variability of the drift rate at s = 1, as it is implemented in JAGS^13^. Figure 1D shows a graphical model representation of the HDM; the full JAGS model code can be downloaded from the Open Science Framework: https://osf.io/zqnbt/.

#### Statistical inference for the hierarchical diffusion model

To estimate best-fitting parameters, all models were fitted using Markov Chain Monte Carlo (MCMC) as implemented in JAGS^35^ with the ‘matjags’ interface (https://github.com/msteyvers/matjags) for MATLAB 2017b (The MathWorks, Inc., Natick, MA). For each model, we ran three chains with a burn-in period of 2000 samples, and 2000 further samples were then retained for analysis. Chain convergence was assessed via the $\widehat{R}$ statistic, where we considered $\widehat{R}$ < 1.1 as an acceptable value^36^. To examine differences in the parameters of interest, we examined the 95% highest density interval (HDI, the smallest region of the posterior that contains the 95% proportion of its mass). If the HDI does not contain 0, there is a 95% probability that the parameter is not 0^15^.

## Results

### Pre-analysis data exclusion

One participant was excluded from the analysis due to a programming error. Two participants with word categorization accuracy less than 90% were excluded from the analysis. Those participants categorized 68.4% and 69.6%, respectively, of the words correctly, while others had 97.2% correct, and we assumed that those two outliers did not work seriously on the task. Eight individuals were rejected because they responded as having perceived something other than CFS in many trials, resulting in less than 30 trials per condition. Data from the remaining 20 participants were analyzed, and of the total trials, 7.4% were excluded − 4.9% due to outlier RTs and 2.5% due to prime perception.

### Reaction time analyses

**Figure 3:**
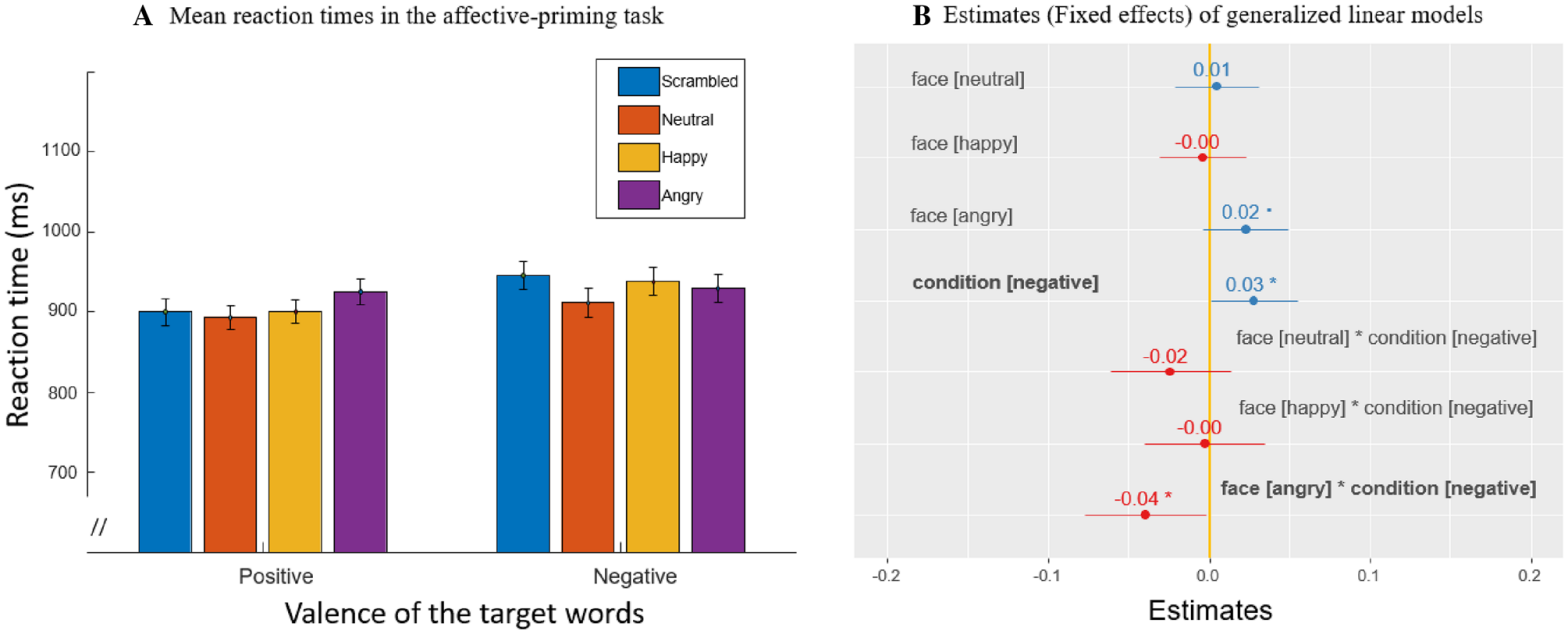
Summary of reaction times (**A**) and estimates of the generalized mixed-effect regressions (**B**) for the affective priming task. The estimate indicates how much the reaction time increases with the fixed effect. The “neutral” line, the vertical intercept that indicates no effect, is drawn in yellow. Significant effects are shown in bold. *p < 0.05.

The generalized linear mixed-effects analysis of the RTs revealed nonsignificant main effects of target word valence (χ^2^(1) = 2.96, p = 0.085) and faces (χ^2^(3) = 1.23, p = 0.743) and a nonsignificant interaction between the two factors (χ^2^(3) = 5.76, p = 0.123) (see Table 1 and Fig. 3 for the RT results). Planned comparisons, which were directly encoded in the model, showed that there was a significant interaction effect between the angry face prime and the negative target word (Fig. 3B; [t (5911) =  − 2.066, p = 0.038]); the RT difference between positive and negative target words was significantly decreased when primed by the angry face versus the scrambled face. This interaction was due to the ‘positive bias’ of scrambled face priming. When primed with invisible scrambled faces, the response times were significantly faster with the positive target word than with the negative target word (Fig. 3B; [t (5911) = 2.079, p = 0.037]); however, the effect was the opposite when primed with the angry face.

**Table 1:** Mean RTs (ms), accuracy (%) and standard deviations (SD) for the affective priming task.

	Positive word	Negative word
**RTs (SD)**		
Scrambled	900 (0.433)	945 (0.496)
Neutral	893 (0.401)	911 (0.467)
Happy	900 (0.410)	938 (0.472)
Angry	926 (0.438)	930 (0.475)
**Accuracy (SD)**		
Scrambled	0.974 (0.034)	0.955 (0.033)
Neutral	0.963 (0.035)	0.966 (0.048)
Happy	0.982 (0.029)	0.989 (0.017)
Angry	0.971 (0.033)	0.978 (0.021)

In summary, the analysis of the RTs suggested that subliminal angry face enhanced judgment when paired with negative words (emotionally congruent conditions). No other faces, including the happy and neutral faces, showed statistical significance. In addition, ‘positive bias’ was observed in the scrambled face prime conditions.

### Hierarchical diffusion model analyses

#### Assessment of convergence and model fit

The $\widehat{R}$ statistic was below 1.01 for all variables, indicating convergence of the MCMC chains to stationary posterior distributions. The correlation between empirical data and the model’s predicted RT quantiles ranged from r = 0.95 to 0.98 for the 8 (4-by-2) conditions (Fig. S1).

#### Model parameter analysis of posterior estimates

**Figure 4:**
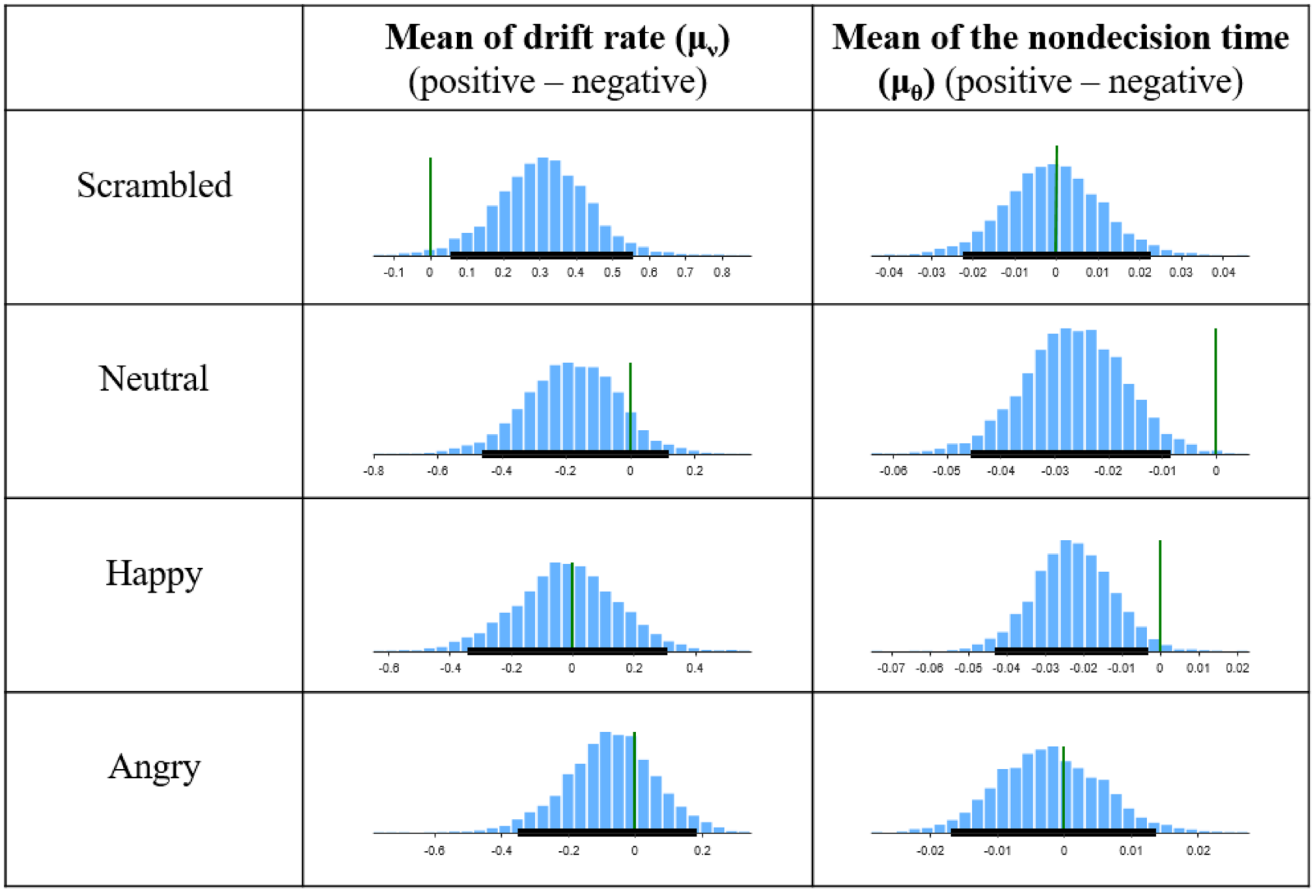
The posterior estimate differences in the mean of the drift rate and the mean of the nondecision time per conditions.

Summary statistics for the drift rate (μ_ν_) and the mean of the nondecision time (μ_θ_) per condition are given in Table 2. The scrambled face prime showed a significantly higher drift rate when categorizing the positive target word than the negative target word (Fig. 4; first row, left column). However, this effect (the positive target word had a higher drift rate than the negative target words) was not observed with any of the three nonscrambled face primes (Fig. 4; 2nd, 3rd, and 4th rows, left column). In other words, a significant decrease in the drift rate was observed only in the scrambled face priming (i.e., the HDI did not contain 0). We further directly compared the change in the drift rate across the word valence conditions by including facial identity in the prime (i.e., Drift rate change in the scrambled face − (Drift rate change in the angry face + Drift rate change in the neutral face + Drift rate change in the happy face)/3). This contrast also showed a significant change in the drift rate under the scrambled face condition (Fig. S2A). In contrast, the means of the nondecision time (μ_θ_) were significantly increased between target word valences, when primed with happy or neutral faces (Fig. 4; 2, and 3rd row, right column), which was not observed with the angry and scrambled face primes. We further investigated the nondecision time difference between conditions, and only the ‘Happy, negative’ condition was greater than the ‘Angry, negative’ condition (HDI = [0.003 0.040]), indicating that a happy face interfered with judging negative words via an increase in the nondecision time.

**Table 2:** Posterior estimates (mean and highest density interval) for the mean of the nondecision time and drift rate.

	Drift rate		Nondecision time	
	Positive	Negative	Positive	Negative
Scrambled	2.65 [2.48 2.86]	2.40 [2.19 2.62]	0.46 [0.44 0.47]	0.46 [0.44 0.48]
Neutral	2.51 [2.29 2.72]	2.68 [2.46 2.90]	0.45 [0.43 0.47]	0.48 [0.46 0.49]
Happy	2.79 [2.53 3.03]	2.82 [2.57 3.09]	0.46 [0.45 0.48]	0.49 [0.47 0.50]
Angry	2.54 [2.33 2.71]	2.64 [2.46 2.84]	0.46 [0.45 0.48]	0.47 [0.45 0.48]

## Discussion

We conducted an experiment to determine how the two types of facial information (facial identity and emotion) affect subsequent decisions using subliminal face primes with positive and negative target words. Our RT analysis results revealed that the interaction between the angry face and scrambled face primes was significant. The significant interaction was mainly due to the ‘positivity bias’ effect^37^, i.e., a processing advantage of positive stimuli over negative stimuli, that was observed in the scrambled face priming condition. However, no significant differences were observed for the happy and neutral face primes. This result is partially consistent with previous studies that reported an affective priming effect using CFS. Experiments using CFS have consistently shown that negative emotional expressions (i.e., fearful or angry faces) gain privileged access to visual awareness over faces with neutral or happy expressions, an effect that is sometimes referred to as “fear advantage”^38–40^. However, since an angry face conveys information on both facial identity and emotion compared to a scrambled face, it is difficult to fully dissociate and understand which type of information is responsible for the differences in RT observed with the angry face prime and the lack of difference observed with the neutral and happy face primes.

We subsequently used the HDM to see how the underlying cognitive processes mapped onto different parameters. In the scrambled face priming condition, the mean of the drift rate was significantly higher with the positive target words. However, this change in drift rate commonly disappeared with all three nonscrambled faces (Fig. 4, left column), resulting in the disappearance of a ‘positive bias’. Since nonscrambled faces commonly have ‘facial identity’ whereas scrambled faces do not, this result suggests that the priming effect of a relatively higher drift rate in the negative condition may be related to facial identity. The change in the drift rate between the word valence conditions elicited by including facial identity in the prime also showed a significant change in the drift rate (Fig. S2A). This result partially supports our hypothesis. In the Introduction, we mentioned that the drift rate (i.e., the rate of evidence accumulation) has a semantic, associative relation. We proposed that, in contrast to the scrambled faces, all three intact faces have facial identity in common and that they would have an associative effect with personality adjectives; thus, the association would facilitate the tasked person’s judgement and would be mapped onto the drift rate parameter. What was unexpected is that the facilitation was only observed for negative target words (Fig. S2B), implying that facial identity only enhances the drift rate of negative target words, thus erasing the ‘positive bias’. In other words, the subliminal facial identity information was only processed to create an association with negative valence words. To our knowledge, this phenomenon has not been reported previously, and further study would be needed to solidify this result.

We have also hypothesized that nondecision time (i.e., short nondecision time provides a head start to the decision process) would account for the facial emotional priming effect on the valence categorization. With respect to the nondecision time, when judging negative words, the happy face prime showed the longest nondecision time, followed by the neutral and angry faces (Table 2). This is in the order of emotional valence (positive–neutral–negative). Additionally, only the happy and neutral face primes showed significant increases in nondecision times (Fig. 4, right column), thereby showing an emotional incongruent effect. This result implied that the emotional information in the facial expression was reflected in the nondecision time. The nondecision time represents the encoding at the stimulus stage; thus, the emotional content in the face provides a head start to the decision process.

In summary, we provide evidence that both facial identity information and emotional information from face priming are processed under CFS. Our result from the simple RT analysis replicated the results of previous studies—only angry faces showed a priming effect. By using the diffusion model, we further discovered that the two types of information in the emotional face were preserved under CFS. This is a main advantage of diffusion model analysis in that it allows the experimenter to distinguish the processes underlying conditions with similar RTs^12^.

This study has several limitations. First, the sample size was relatively small, and the ability to draw generalizations with statistical significance is limited. However, we conducted a power analysis and an additional power calculation using PANGEA (http://jakewestfall.org/pangea/), and this showed that our data have sufficient power to demonstrate the priming effect^41^. As discussed earlier, we used HDM, which is ideally suited to handle data sets with few trials per participant, to estimate parameters to obtain a reliable measurement of parameters for each condition. Second, the present study did not elucidate the neural mechanism that is responsible for affective priming. In future studies, it will be necessary to investigate the neural mechanisms underlying the generation of affective response bias using electroencephalography or functional MRI. Third, we did not collect or match the arousal ratings of the target words. It has been reported that the valence and arousal of Korean adjectives are highly negatively correlated (r = − 0.57)^42^. Based on that report, our simulation of arousal ratings indicates a low chance of significantly different arousal. However, although we matched the valence, the arousal associated with specific words may confound the results since arousal by the target might possibly have influenced the valence judgment. Fourth, we did not counterbalance the response-mapping of “negative” and “positive” responses on the direction keys. Fifth, in this task, we excluded the perceived trials based on the participants’ subjective reports. Some existing objective tasks (i.e., two-alternative forced choice (2AFC) discrimination task) could improve the reliability of the results^6,43^. Sixth, although we used grayscale, elliptical, low-contrast primes, the differences in race between the participants and the facial primes may limit the generalizability of this study^44,45^. Seventh, we only analyzed RT and did not conduct an analysis of the error rates, as in previous studies on similar topics^2,6^. Since we have made our data available to the public, anyone who is interested can investigate.

## Supplementary Information

Supplementary Figures.

## Data Availability

The data and the code used in the reported analyses are available for downloading from the Open Science Framework: https://osf.io/zqnbt/.

## References

[1] Roberts ID, Hutcherson CA (2019). Affect and decision making: Insights and predictions from computational models. Trends Cogn. Sci..

[2] Almeida J, Pajtas PE, Mahon BZ, Nakayama K, Caramazza A (2013). Affect of the unconscious: Visually suppressed angry faces modulate our decisions. Cogn. Affect. Behav. Neurosci..

[3] Fazio RH (2001). On the automatic activation of associated evaluations: An overview. Cogn. Emot..

[4] Anderson E, Siegel E, White D, Barrett LF (2012). Out of sight but not out of mind: Unseen affective faces influence evaluations and social impressions. Emotion.

[5] Lapate RC, Rokers B, Li T, Davidson RJ (2014). Nonconscious emotional activation colors first impressions: A regulatory role for conscious awareness. Psychol. Sci..

[6] Yang Y-H, Yeh S-L (2018). Unconscious processing of facial expression as revealed by affective priming under continuous flash suppression. Psychon. Bull. Rev..

[7] Yuan J, Hu X, Lu Y, Bodenhausen GV, Fu S (2017). Invisible own-and other-race faces presented under continuous flash suppression produce affective response biases. Conscious. Cogn..

[8] Tsuchiya N, Koch C (2005). Continuous flash suppression reduces negative afterimages. Nat. Neurosci..

[9] Tsuchiya N, Koch C, Gilroy LA, Blake R (2006). Depth of interocular suppression associated with continuous flash suppression, flash suppression, and binocular rivalry. J. Vis..

[10] Mudrik L, Faivre N, Koch C (2014). Information integration without awareness. Trends Cogn. Sci..

[11] Ratcliff R (1978). A theory of memory retrieval. Psychol. Rev..

[12] Voss A, Nagler M, Lerche V (2013). Diffusion models in experimental psychology: A practical introduction. Exp. Psychol..

[13] Wabersich D, Vandekerckhove J (2014). Extending JAGS: A tutorial on adding custom distributions to JAGS (with a diffusion model example). Behav. Res. Methods.

[14] Almeida J (2014). Grasping with the eyes: The role of elongation in visual recognition of manipulable objects. Cogn. Affect. Behav. Neurosci..

[15] Vandekerckhove J, Tuerlinckx F, Lee MD (2011). Hierarchical diffusion models for two-choice response times. Psychol. Methods.

[16] Wabersich, D. & Vandekerckhove, J. The RWiener package: An R package providing distribution functions for the wiener diffusion model. *R J.* **6** (2014).

[17] Lee MD, Wagenmakers E-J (2014). Bayesian Cognitive Modeling: A Practical Course.

[18] Posamentier MT, Abdi H (2003). Processing faces and facial expressions. Neuropsychol. Rev..

[19] Bruce V, Young A (1986). Understanding face recognition. Br. J. Psychol..

[20] Jiang Y (2009). Dynamics of processing invisible faces in the brain: Automatic neural encoding of facial expression information. Neuroimage.

[21] Jiang Y, He S (2006). Cortical responses to invisible faces: Dissociating subsystems for facial-information processing. Curr. Biol..

[22] Haxby JV, Hoffman EA, Gobbini MI (2000). The distributed human neural system for face perception. Trends Cogn. Sci..

[23] Voss A, Rothermund K, Gast A, Wentura D (2013). Cognitive processes in associative and categorical priming: A diffusion model analysis. J. Exp. Psychol. Gen..

[24] Houwer JD, Hermans D, Rothermund K, Wentura D (2002). Affective priming of semantic categorisation responses. Cogn. Emot..

[25] Todorova L, Neville DA (2020). Associative and identity words promote the speed of visual categorization: A hierarchical drift diffusion account. Front. Psychol..

[26] Yap MJ, Balota DA, Tan SE (2013). Additive and interactive effects in semantic priming: Isolating lexical and decision processes in the lexical decision task. J. Exp. Psychol. Learn. Mem. Cogn..

[27] Hesselmann G, Darcy N, Ludwig K, Sterzer P (2016). Priming in a shape task but not in a category task under continuous flash suppression. J. Vis..

[28] Faul F, Erdfelder E, Lang A-G, Buchner A (2007). G* Power 3: A flexible statistical power analysis program for the social, behavioral, and biomedical sciences. Behav. Res. Methods.

[29] Lundqvist D, Flykt A, Öhman A (1998). The Karolinska directed emotional faces (KDEF). CD ROM Dept. Clin. Neurosci. Psychol. Sect. Karolinska Inst..

[30] Kim H-W, Kim C-Y, Blake R (2017). Monocular perceptual deprivation from interocular suppression temporarily imbalances ocular dominance. Current Biology.

[31] Lo S, Andrews S (2015). To transform or not to transform: Using generalized linear mixed models to analyse reaction time data. Front. Psychol..

[32] Bates, D., Maechler, M., Bolker, B. & Walker, S. lme4: Linear mixed-effects models using Eigen and S4. R package version 1.0-4. https://cran.r-project.org/web/packages/lme4/index.html (2013).

[33] Team, R. C. R: A language and environment for statistical computing. (2013).

[34] Fox J, Weisberg S (2018). An R Companion to Applied Regression.

[35] Plummer, M. in *Proceedings of the 3rd international workshop on distributed statistical computing.* 1–10 (Vienna, Austria).

[36] Gelman A, Hill J (2006). Data Analysis Using Regression and Multilevel/Hierarchical Models.

[37] Kauschke C, Bahn D, Vesker M, Schwarzer G (2019). The role of emotional valence for the processing of facial and verbal stimuli–positivity or negativity bias?. Front. Psychol..

[38] Tsuchiya N, Moradi F, Felsen C, Yamazaki M, Adolphs R (2009). Intact rapid detection of fearful faces in the absence of the amygdala. Nat. Neurosci..

[39] Yang E, Zald DH, Blake R (2007). Fearful expressions gain preferential access to awareness during continuous flash suppression. Emotion.

[40] Hedger N, Adams WJ, Garner M (2015). Fearful faces have a sensory advantage in the competition for awareness. J. Exp. Psychol. Hum. Percept. Perform..

[41] Westfall, J. *PANGEA: Power ANalysis for GEneral Anova designs*. Unpublished manuscript. Available at http://jakewestfall.org/publications/pangea.pdf (2015).

[42] Hong Y, Nam Y-E, Lee Y (2016). Developing Korean affect word list and it’s application. Korean J. Cogn. Sci..

[43] Lin, Z. & He, S. Seeing the invisible: The scope and limits of unconscious processing in binocular rivalry. *Nat. Proc.* 1–1 (2008). 10.1016/j.pneurobio.2008.09.002 PMC2689366 18824061

[44] Sauter DA, Eisner F (2013). Commonalities outweigh differences in the communication of emotions across human cultures. Proc. Natl. Acad. Sci..

[45] Jack RE, Garrod OG, Yu H, Caldara R, Schyns PG (2012). Facial expressions of emotion are not culturally universal. Proc. Natl. Acad. Sci..

