# Supplementary material for: Diffusion model-based understanding of subliminal affective priming in continuous flash suppression
Source: Sci Rep. 2021 Jun 1;11:11534. doi: 10.1038/s41598-021-90917-w (PMC8169665; doi:10.1038/s41598-021-90917-w)
Supplement: Supplementary file 1 — Supplementary Figures. [file 41598_2021_90917_MOESM1_ESM.pdf]

Diffusion model-based understanding of subliminal affective priming in continuous flash suppression

Author names: Minchul Kim, MD[1,2], Jeeyeon Kim[1],

Jaejoong Kim, MD, PhD[1], Bumseok Jeong, MD, PhD[1]

Author affiliations:

[1] Graduate School of Medical Science and Engineering (GSMSE), Korea Advanced Institute of Science and Technology (KAIST), Daejeon, South Korea

[2] Department of Radiology, Kangbuk Samsung Hospital, Sungkyunkwan University School of Medicine, Seoul, South Korea

Corresponding author:

Bumseok Jeong, M.D., Ph.D.

Graduate School of Medical Science and Engineering (GSMSE), Korea Advanced Institute of Science and Technology (KAIST)

291 Daehak-ro, Yuseong-gu, Daejeon, 34141, Republic of Korea

Tel: +82-42-350-4285

E-mail: bs.jeong@kaist.ac.kr

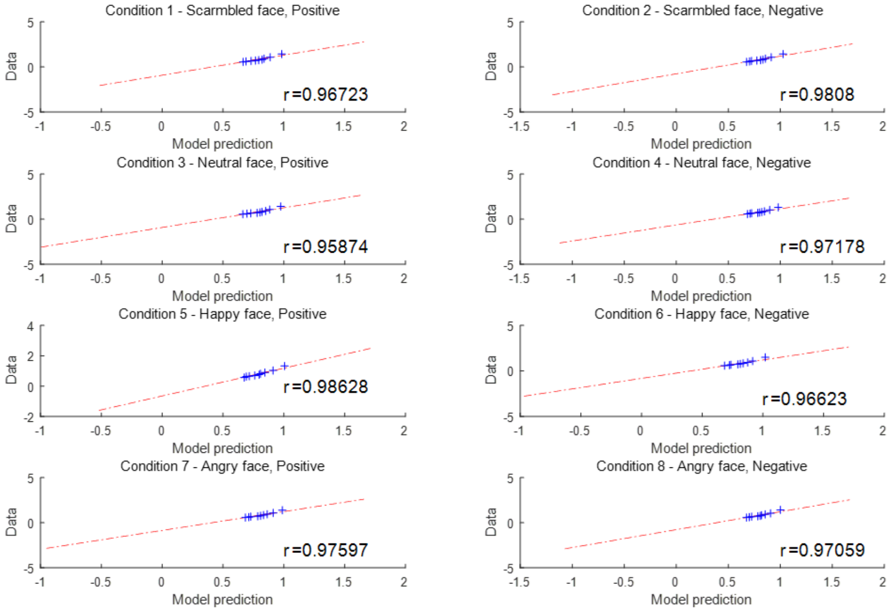

Figure S1. Plot showing group RT quantiles (q = 0.1, 0.2, 0.3, 0.4, 0.5, 0.6, 0.7, 0.8, 0.9) based on empirical data and model prediction for each condition. Simulations of RT distributions for the affective priming task showed that the model fitted our data very well, as the correlation between the empirical and model RT quantiles was greater than r = 0.958. We coded error response as negative RT.

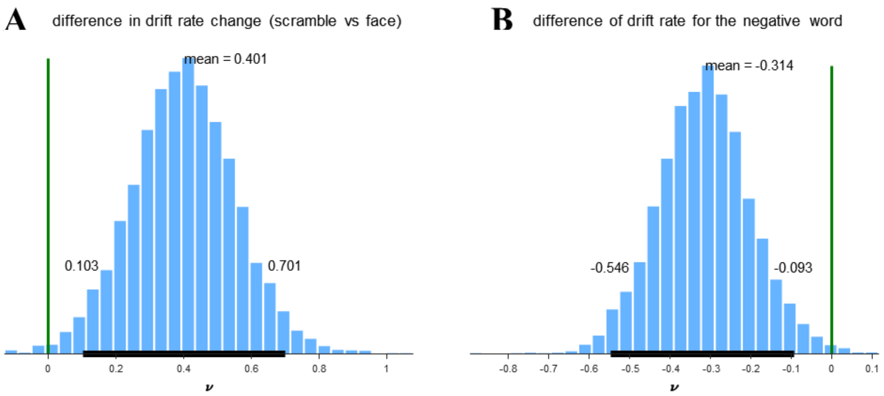

Figure S2. A. Difference in drift rate change between target word valence (negative versus positive) between scrambled and non-scrambled faces that have facial identity in common. The contrast is measured as : Drift rate change in the scrambled face – (Drift rate change in the angry face + Drift rate change in the neutral face + Drift rate change in the happy face)/3. **B.** Difference in drift rate in the negative target words condition between scrambled and non-scrambled faces that have facial identity in common. The contrast is measured as : Drift rate in scrambled face with negative target - (Drift rate in angry face with negative target + Drift rate in neutral face with negative target + Drift rate in happy face prime with negative target word)/3.
